# MAPK signaling pathway regulates cerebrovascular receptor expression in human cerebral arteries

**DOI:** 10.1186/1471-2202-14-12

**Published:** 2013-01-23

**Authors:** Saema Ansar, Sajedeh Eftekhari, Roya Waldsee, Elisabeth Nilsson, Ola Nilsson, Hans Säveland, Lars Edvinsson

**Affiliations:** 1 Department of Clinical Sciences, Division of Experimental Vascular Research, Lund University, Lund, Sweden; 2 Department of Neurosurgery, Lund University Hospital, Lund, Sweden

**Keywords:** Human cerebral arteries, Endothelin receptors, Angiotensin receptors, 5-hydroxytryptamine receptors, Thromboxane receptors, Mitogen activated protein kinase

## Abstract

**Background:**

Cerebral ischemia results in enhanced expression of contractile cerebrovascular receptors, such as endothelin type B (ET_B_), 5-hydroxytryptamine type 1B (5-HT_1B_), angiotensin II type 1 (AT_1_) and thromboxane (TP) receptors in the cerebral arteries within the ischemic area. The receptor upregulation occurs via activation of the mitogen-activated protein kinases (MAPK) pathway. Previous studies have shown that inhibitors of the MAPK pathway diminished the ischemic area and contractile cerebrovascular receptors after experimental cerebral ischemia. The aim of this study was to examine if the upregulation of contractile cerebrovascular receptors after 48 h of organ culture of human cerebral arteries involves MAPK pathways and if it can be prevented by a MEK1/2 inhibitor. Human cerebral arteries were obtained from patients undergoing intracranial tumor surgery. The vessels were divided into ring segments and incubated for 48 h in the presence or absence of the specific MEK1/2 inhibitor U0126. The vessels were then examined by using in vitro pharmacological methods and protein immunohistochemistry.

**Results:**

After organ culture of the cerebral arteries the contractile responses to endothelin (ET)-1, angiotensin (Ang) II and thromboxane (TP) were enhanced in comparison with fresh human arteries. However, 5-carboxamidotryptamine (5-CT) induced decreased contractile responses after organ culture as compared to fresh arteries. Incubation with U0126 diminished the maximum contraction elicited by application of ET-1, Ang II and U46619 in human cerebral arteries. In addition, the MEK1/2 inhibitor decreased the contractile response to 5-CT. Immunohistochemistry revealed that organ culture resulted in increased expression of endothelin ET_A_, endothelin ET_B_ angiotensin AT_2,_ 5-hydroxytryptamine 5-HT_1B_ and thromboxane A2 receptors, and elevated levels of activated pERK1/2, all localized to the smooth muscle cells of the cerebral arteries. Co-incubation with U0126 normalized these proteins.

**Conclusion:**

The study demonstrated that there is a clear association between human cerebrovascular receptor upregulation via transcription involving activation of the MAPK pathway after organ culture. Inhibition of the MAPK pathways attenuated the vasoconstriction mediated by ET_,_ AT and TP receptors in human cerebral arteries and the enhanced expression of their receptors. The results indicate that MAPK inhibition might be a novel target for treatment of cerebrovascular disorders.

## Background

Large efforts have during the last few decades been made to understand the intracellular mechanisms involved in ischemia-induced cerebral damage and to develop drugs that protect the brain from damage once a stroke has occurred. However, despite extensive research into genetics and molecular biology associated with cerebral ischemia, few acute therapies have proven effective in the clinic [1]. Investigations have revealed that cerebral ischemia is accompanied by modifications in the expression of genes regulating receptor expressions in cerebrovascular smooth muscle cell (SMC)s associated with the cerebral ischemia [2]. Thus, experimental and clinical studies of cerebral ischemia have reported increased levels of the potent vasoconstrictor substances endothelin (ET) [3,4], 5-hydroxytryptamine (5-HT) [5,6], angiotensin (Ang II) [7] and thromboxane (TXA_2_) [8,9]. ET-1, 5-HT, Ang II and TXA_2_ are all potent vasoconstrictors of cerebral arteries that mediate effects through the family of G-protein coupled receptors (GPCRs) [10,11]; endothelin A (ET_A_), endothelin B (ET_B_) [11,12], 5-HT receptors [5], the angiotensin II type 1 (AT_1_) and type 2 (AT_2_) receptors [13,14] and the thromboxane receptor (TP) [15].

Cerebral ischemia is multifactorial, involves a number of neuronal and glial mechanisms; however, several cerebrovascular receptors are in addition involved in the pathophysiology of cerebral ischemia. There is upregulation (enhanced expression) of contractile ET_B_, 5-HT_1B_, AT_1_ and TP receptors in major cerebral arteries from experimental focal and global ischemia, via enhanced transcription and translation [16-24]. This upregulation of cerebrovascular receptors leads to enhanced vasoconstriction and correlates with reduction in regional cerebral blood flow (rCBF) and degree of neurology deficit [21]. Blockade of the individual subtypes of receptors involved might prevent or reduce the cerebral ischemia to a certain degree; we hypothesize that treatment aimed at a common signaling pathway would be more beneficial by avoiding the administration of several antagonists with circulatory consequences.

The mitogen-activated protein kinase (MAPK) pathways are implicated in neuronal death and survival after stroke. A time study of the alteration in cerebrovascular MAPKs after experimental subarachnoid hemorrhage (SAH) revealed that there was early (within minutes) and sustained activation of the specific extracellular signal-regulated kinases (ERK)1/2 pathway, while the p38 and JNK pathways were activated first at 48 hours [25]. The ERK1/2 pathway can be inhibited at various points upstream such as at ras-raf-MEK1/2; inhibition of this pathway with a specific MEK1/2 (the MAPKK of ERK1/2) inhibitor abolished the receptor upregulation as well as preventing the CBF reduction and diminishes the infarct [16,26,27]. ERK1/2 belongs to the family of MAPK and is phosphorylated and thereby activated by the MAP kinase/ERK kinase (MEK)1/2. Several studies have shown an involvement of the MEK/ERK1/2 signalling pathway in cerebral ischemia [26,28].

Organ culture is an in vitro method for investigating cellular mechanisms involved in upregulation of vasocontractile G-protein coupled receptors. Organ culture is not a model for stroke, however, changes in vasoconstrictor responses after in vitro organ culture show a remarkable similarity to changes observed in animal models of ischemic and hemorrhagic stroke. Thus, there is an upregulation of contractile G-protein receptors after SAH [21] and focal ischemia [22,23] which also is observed in organ culture [29]. This make the organ culture model an appropriate model for investigating the pharmacological characteristics and underlying molecular and cellular mechanism involved in the upregulation of vasocontractile G-protein coupled receptors. The upregulation of contractile receptors in the SMCs are prevented with MAPK inhibitor both in organ culture [29] and experimental stroke [27,30].

In the design of future cerebrovascular therapeutics it is important that the intracellular mechanisms are characterised in human subjects. Here we hypothesize that there is an upregulation of contractile cerebrovascular receptors after 48 h of organ culture in human cerebral arteries and that this upregulation occurs via the MAPK ERK1/2 pathway and can be inhibited by the MEK1/2 inhibitor U0126.

## Methods

All procedures were carried out strictly within national laws and guidelines and approved by the Ethical Committee at the University of Lund (LU-818-01) and has been performed in accordance with the Declaration of Helsinki. A consent was obtained from the participants prior to surgery.

### Tissue collection and organ culture procedure

Cortical arteries were obtained from patients undergoing neurological surgery for brain tumors. The arteries obtained were physiological arteries with surrounded tumor tissue, the arteries were carefully dissected free of connective tissue leaving the vessel with intact intima, media and adventitia. The arteries were immediately immersed in cold sterile Dulbecco’s modified Eagle’s medium (DMEM,Gibco, Invitrogen, Carlsbad, CA, USA) and transported to the laboratory. The arteries were cut into 1-mm long ring segments for *in vitro* pharmacological experiments and 3-mm for immunohistochemistry. The outer diameters were between 300 and 800 μm.

### Organ culture

The arterial segments were cultured for 48 hours at 37°C in humidified 5% CO_2_ and air in Dulbecco’s modified Eagle’s medium supplemented with pencillin (100 U/ml), streptomycin (100 μg/ml) and amphotericin B (25 μg/ml). The method of blood vessel culture has been described previously [31]. The segments were cultured in the absence or presence of the MEK1/2 inhibitors U0126 (5 μM). The selection of the inhibitor U0126 was based on previous detailed work on isolated arteries in organ culture, were U0126 was demonstrated to be the best of all available MEK1/2 inhibitors to inhibit the GPCRs and MAPK pathway [29,32].

### In vitro pharmacology myograph experiments

For contractile experiments a sensitive myograph was used for recording the isometric tension in isolated cerebral arteries [33,34]. The vessels were cut into 1 mm long cylindrical segments and mounted on two 40 μm in diameter stainless steel wires in a Myograph (Danish Myo Technology A/S, Denmark). One wire was connected to a force displacement transducer attached to an analogue-digital converter unit (ADInstruments, Oxford, UK). The other wire was connected to a micrometer screw, allowing fine adjustments of vascular tone by varying the distance between the wires. Measurements were recorded on a computer by use of a PowerLab unit (ADInstruments). The segments were immersed in a temperature controlled buffer solution (37°C) of the following composition (mM) NaCl 119, NaHCO_3_ 15, KCl 4.6, MgCl_2_ 1.2, NaH_2_PO_4_ 1.2, CaCl_2_ 1.5 and glucose 5.5. The buffer was continuously aerated with oxygen enriched with 5% CO_2_ resulting in a pH of 7.4. Initially, the vessel segments were normalized and set to an initial resting tone of 2 mN that is the tone that it would have if relaxed and under a transmural prerssure of 100 mmHg. The vessels were allowed to stabilize at this tone for 1 hour. The contractile capacity was determined by exposing the vessels to an isotonic solution containing 63.5 mM of K^+^, obtained by partial change of NaCl for KCl in the above buffer. The contraction induced by K^+^ was used as reference for the contractile capacity [34]. Only vessels responding by contraction of at least 2 mN to potassium were included in the study.

Concentration-response curves were obtained by cumulative application of 5-carboxamidotryptamine (5-CT; specific 5-HT_1_ receptor agonist (Sigma, St. Louis, USA)) in the concentration range 10 ^–12^ to 10 ^–5^ M, ET-1 (Endothelin ET_A_ and ET_B_ receptor agonist (AnaSpec, San Jose, USA)) in the concentration range 10 ^–14^ to 10 ^–7^ M, U46619 (Thromboxane A2 receptor agonist (Sigma, St. Louis, USA)) in the concentration range 10 ^–12^ to 10 ^–6^ M and Ang II (Angiotensin AT_1_ and AT_II_ receptor agonist (Sigma, St. Louis, USA)) in the concentration range 10 ^–12^ to 10 ^–6^ M.

### Immunohistochemistry

**Figure 1:**
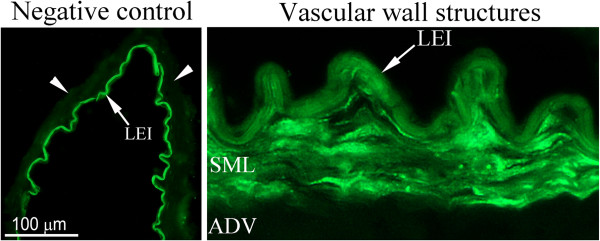
**Negative control; omission of the primary antibody or only the primary antibody applied.** No immunoreactivity is detected within the smooth muscle cell layer (arrow points). Only auto-fluorescence in lamina elastica interna (arrow) is detected. LEI; lamina elastica interna. Vascular wall structures; example of immunohistochemical staining on human artery showing the different wall structures. ADV; adventitial layer, LEI; lamina elastica interna, SML; smooth muscle cell layer.

For immunofluorescence the cerebral artery segments were embedded in Tissue TEK (Gibo, Invitrogen A/S, Taastrup, Denmark), frozen at -80°C and subsequently sectioned into 10 μm thick slices. Cryostat sections were fixed for 10 minutes in ice-cold acetone (−20°C) and thereafter rehydrated in phosphate buffered-saline (PBS, pH 7.2) containing 0.25% Triton X-100 (PBST), for 3×5 minutes. The sections were then permeabilized and blocked for 1 h in blocking solution containing PBS and 5% normal donkey serum and then incubated over night at 4°C with either of the following primary antibodies; rabbit anti ET_A_ (1:50, Santa Cruz Biotechnology, CA, U.S.A., sc-33535), rabbit anti ET_B_ (1:200, Abcam, Cambridge, UK, ab1921), rabbit anti AT_1_ (1:100, Santa Cruz Biotechnology, sc-1173), AT_2_ (1:100, Santa Cruz Biotechnology, sc-9040), 5-HT1_B_ (1:100, Santa Cruz Biotechnology, sc-1460), rabbit TP-receptor (1:100, Cayman Chemical company, Michigan, U.S.A., 10004452) and rabbit anti phospho-ERK p44/42 MAPK (1:50, Cell Signaling Technology, Beverly, CA, U.S.A., #4376). The primary antibodies were diluted in PBST, 1% bovine serum albumin (BSA) and 3% normal donkey serum. On the second day sections were rinsed in PBST for 3×15 minutes and incubated with the secondary antibody (1 h, room-temperature). The secondary antibody used was Cy™^2^ conjugated donkey anti rabbit (1:200, Jackson ImmunoResearch, West Grove, PA, U.S.A, 711-165-152) diluted in PBST and 1% BSA. The sections were washed subsequently with PBST and mounted with Crystal mounting medium (Sigma, St.Louis, MO, U.S.A). Immunoreactivity was visualized with an Olympus Microscope (BX 60, Japan) at the appropriate wavelength. Negative controls for all antibodies were made by omitting primary antibodies. In all cases, no specific staining was found; only auto-fluorescence in lamina elastica interna was seen (Figure 1). To evaluate the auto-fluorescence in lamina elastica interna, controls were made with only primary antibodies.

#### Calculations and statistics

Data are expressed as mean ± standard error of the mean (s.e.m.), and n refers to the number of patients. Statistical analyses were performed with Kruskal-Wallis non-parametric test with Dunn’s post-hoc test, where P<0.05 was considered significant.

### In vitro pharmacology

Contractile responses in each segment are expressed as percentage of the 63.5 mM K^+^ induced contraction. E_max_ value represents the maximum contractile response elicited by an agonist and the pEC_50_ the negative logarithm of the drug concentration that elicited half the maximum response.

### Immunohistochemistry

Measurements were made in order to quantify the immunoreactivity of the protein expressions in the different groups. The evaluation of receptors and pERK expression was performed by measurements of the fluorescence intensity using the software image J http://rsb.info.nih.gov/ij/. The immunoreactivity of the individual receptors was visualized with the same microscope settings during the same day for all groups. The intensity measurements were performed in a blinded manner. The fluorescence intensity was measured in four given areas in the smooth muscle layer of each sample (always clockwise). Values were given by dividing the fluorescence intensity to measured area of each sample. These values are presented as percentage fluorescence in the cultured groups compared to the fresh groups (henceforth only mentioned as control), where the fresh (control) group is set to 100%.

## Results

### Functional in vitro pharmacology

K^+^ -induced contraction, E_max_, and pEC_50_ values for respective group are presented in Table 1. Of all vessels tested 72% responded to a K^+^ -induced contraction.

**Table 1:** Contractile effects of ET-1, U46619, Ang II and 5-CT in human cerebral arteries

	**N**	**K**^**+ **^**mean ± s.e.m**	**E**_**max **_**(%) ± s.e.m**	**pEC**_**50 **_**± s.e.m**
**ET-1**				
Control	6	6.76 ± 1.32	107 ± 12^a^	7.20 ± 0.89
Organ Culture	6	4.37 ± 1.21	143 ± 22 ^a,b^	8.74 ± 0.25
Organ Culture + U0126	7	3.53 ± 0,90	57 ± 8 ^b^	8.49 ± 0,10
**U46619**				
Control	7	8.26 ± 1.98	102 ± 15^a^	7.35 ± 0.74
Organ Culture	7	5.10 ± 1.60	141 ± 11^a,b^	7.18 ± 0.18
Organ Culture + U0126	4	3.65 ± 1.89	76 ± 40^b^	6.64 ± 0.10
**Ang II**				
Control	6	1.67 ± 0.54	12 ± 2^a^	
Organ Culture	5	4.44 ± 1.92	43 ± 12^a,b^	9.15 ± 0.82
Organ Culture + U0126	4	4.46 ± 2.41	1 ± 1^b^	9.05 ± 1.13
**5-CT**				
Control	6	6.76 ± 1.36	50 ± 8	7.45 ± 0.66
Organ Culture	4	3.90 ± 1.59	17 ± 5^b^	7.35 ± 1.21
Organ Culture + U0126	7	3.53 ± 0.90	2 ± 1^b^	7.38 ± 0.73

Responses were characterized by E_max_ values, expressed as percent of 63 mM K^+^ -induced contraction, and pEC_50_ values (negative logarithm of the molar concentration that produces half maximum contraction). Values are represented as mean ± s.e.m and n represents number of patients. ^a^ = significant difference between organ culture and control groups, ^b^ = significant difference between organ culture and organ culture treated with U0126.

### ET-1 receptor

#### Contractile response to ET-1

**Figure 2:**
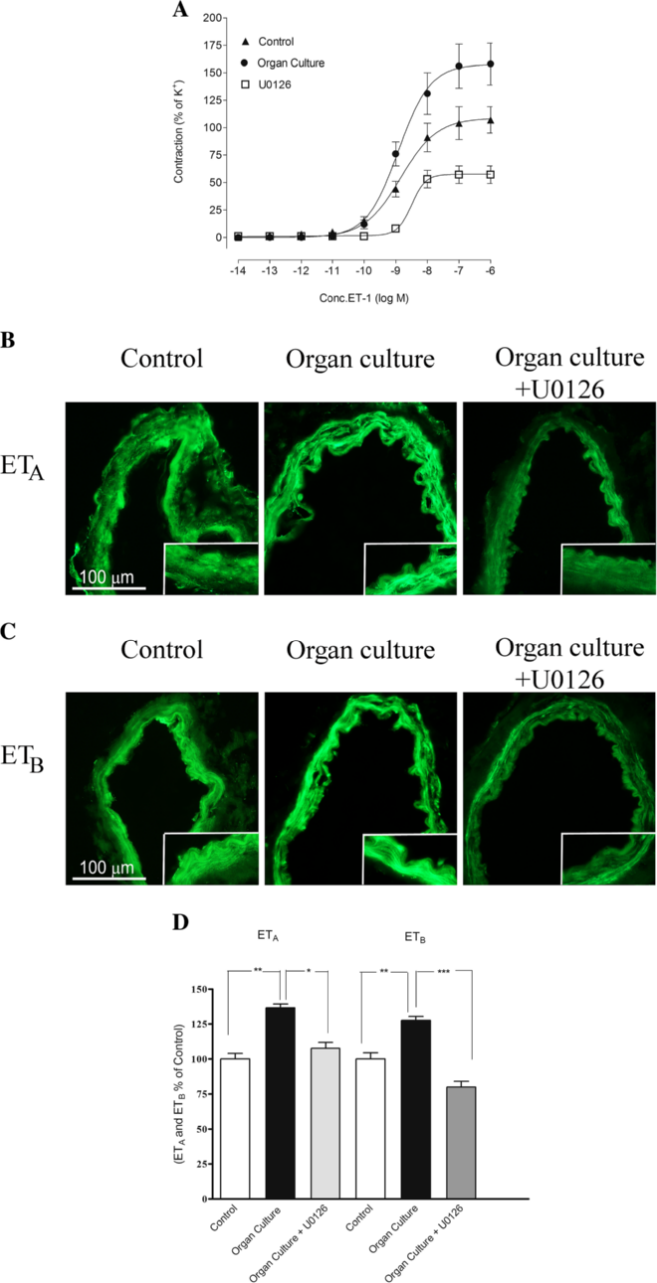
**A. Concentration response curves elicited by cumulative application of ET-1 in human cerebral arteries incubated for 48 h with or without the MEK1/2 inhibitor U0126 (5 μM) and control human arteries.** The responses to ET-1 are clearly increased in the incubated arteries as compared to control arteries. The enhanced contraction observed after organ culture was inhibited in arteries cultured with U0126. Data are expressed as mean ± s.e.m and n refer to the number of patients used. **B** and **C** Sections from the human cerebral artery showing ET_A_ and ET_B_ receptor immunoreactivity in the smooth muscle cell layer. **D** Expression of ET_A_ and ET_B_ protein levels in human cerebral arteries incubated for 48 h with or without the MEK1/2 inhibitor U0126 (5 μM) and control human arteries. Data are expressed as percentage of control and given as mean ± s.e.m. *P<0.05, ** *P*<0.01.

In cultured arteries ET-1 yielded contractions with an E_max_ of 143 ± 22% and a pEC_50_ of 8.74 ± 0.25. These values were significantly higher than those observed in control arterial segments, in which an E_max_ of 107 ± 12% was observed. This is in accordance with previous results, which show a similar upregulation in human cerebral arteries after organ culture [35]. The presence of U0126 during the organ culture produced a significantly attenuated ET-1 induced response, with an E_max_ of 57 ± 8% compared to the cultured arteries (Figure 2A, Table 1).

#### Protein expression examined by immunohistochemistry

The ET_A_ receptor protein was increased after organ culture (137 ± 3%) as compared to control (100 ± 4%). Incubation with U0126 prevented the increased expression of ET_A_ (107 ± 4%) receptor protein on the smooth muscle cells (Figure 2B and 2D, Table 2). In addition, the ET_B_ receptor protein was expressed in the smooth muscle cells and this signal was increased in organ culture (128 ± 3%) as compared to control arteries (100 ± 6%). Treatment with the MEK1/2 inhibitor U0126 prevented the upregulation of ET_B_ (80 ± 4%), receptor protein levels in the smooth muscle cell layer as compared to the organ culture (Figure 2C, 2D Table 2).

**Table 2:** Receptor protein levels in human cerebral arteries

	**Control**	**Organ.Culture**	**Organ Culture + treatment with U0126**
ET_B_ (%) ± s.e.m	100 ± 6 ^a^	128 ± 3 ^a, b^	80 ± 4 ^b^
ET_A_ (%) ± s.e.m	100 ± 4 ^a^	137 ± 3 ^a, b^	107 ± 4 ^b^
TP (%) ± s.e.m	100 ± 3	119 ± 5	106 ± 3
AT_1_ (%) ± s.e.m	100 ± 2 ^a^	62 ± 4 ^a^	82 ± 7
AT_2_ (%) ± s.e.m	100 ± 2 ^a^	120± 2 ^a^	111 ± 1
5-HT_1B_ (%) ± s.e.m	100 ± 6 ^a^	128 ± 3 ^a, b^	84 ± 4 ^b^
pERK1/2 (%) ± s.e.m	100 ± 2 ^a^	128 ± 2 ^a,b^	86 ± 2 ^b^

Activation of the different protein levels measured with immunohistochemistry in human arteries. Values are expressed as percentage of control and given as mean ± s.e.m. ^a^ = significant difference between organ culture and control groups, ^b^ = significant difference between organ culture and organ culture treated with U0126.

### Thromboxane receptor

#### Contractile response

**Figure 3:**
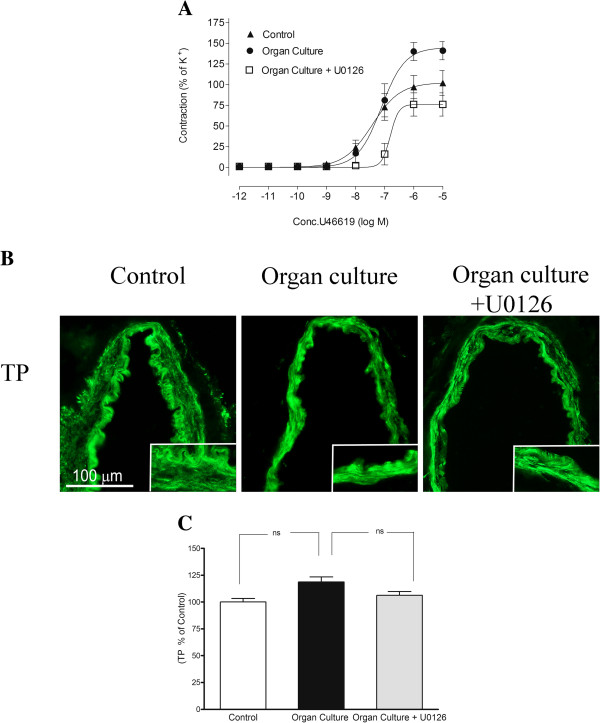
**A. Concentration response curves elicited by cumulative application of U46619 in human cerebral arteries incubated for 48 h, human cerebral arteries incubated for 48 h with the MEK1/2 inhibitor U0126 (5 μM) and control human arteries.** Data are expressed as mean ± s.e.m. **B**. Sections from the human cerebral artery showing TP receptor immunoreactivity in the smooth muscle cell layer. **C**. Expression of TP protein levels in human cerebral arteries incubated for 48 h with or without the MEK1/2 inhibitor U0126 (5 μM) and control human arteries. Data are expressed as percentage of fresh and given as mean ± s.e.m. *P<0.05, ** *P*<0.01.

In cultured arteries U46619 yielded contractions with an E_max_ of 141 ± 11%. This value was significantly higher than those observed in control arterial segments, in which an E_max_ of 102 ± 15% was observed (Figure 3A). The presence of the MEK1/2 inhibitor U0126 during the organ culture produced a significantly attenuated U46619 contractile response, compared to the cultured arteries. There was no significant difference in the E_max_ between control arteries and cultured arteries treated with U0126 (Figure 3A, Table 1).

#### Protein expression examined by immunohistochemistry

The TP receptor protein was expressed in the smooth muscle cells and this signal was slightly increased in organ culture (119 ± 5%) as compared to control arteries (100 ± 3%). Treatment with the MEK1/2 inhibitor U0126 prevented the upregulation of TP (106 ± 3%), receptor protein levels in the smooth muscle cell layer as compared to the organ culture, however not significantly (Figure 3B-C and Table 2).

### Angiotensin receptor

#### Contractile response to Ang II

**Figure 4:**
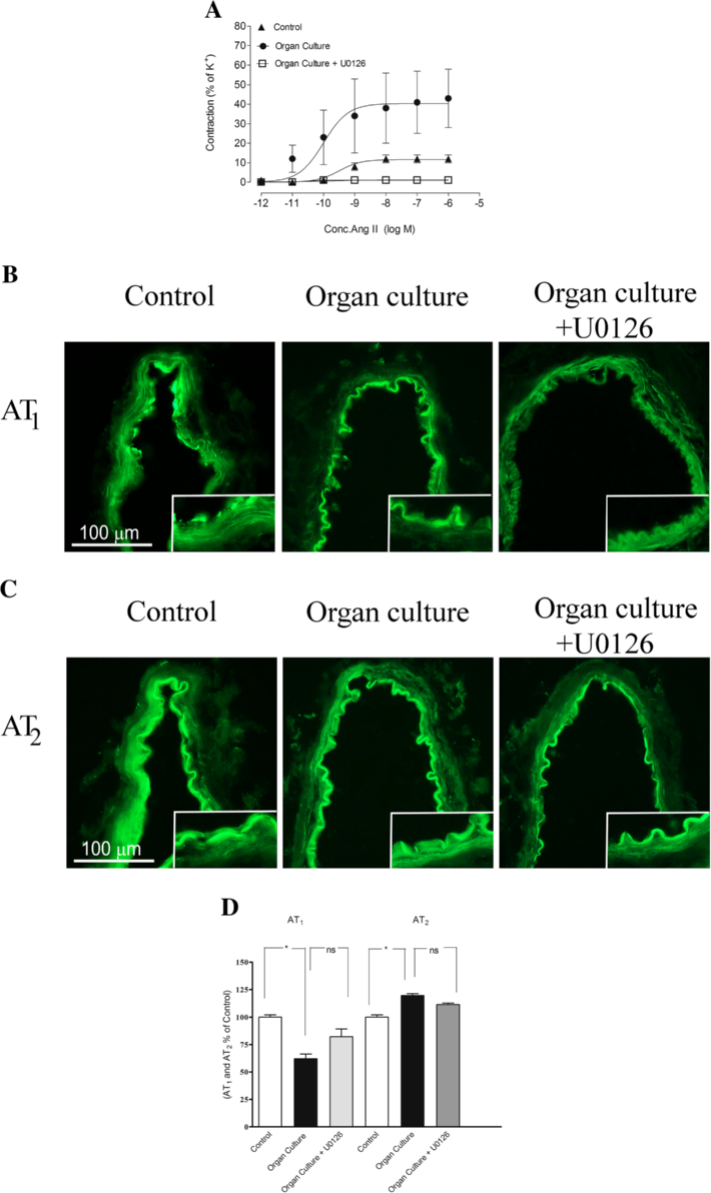
**A. Concentration response curves elicited by cumulative application of Ang II in human cerebral arteries incubated for 48 h, human cerebral arteries incubated for 48 h with the MEK1/2 inhibitor U0126 (5 μM) and control human arteries.** Data are expressed as mean ± s.e.m. **B** and **C**. Sections from the human cerebral artery showing AT_1,_ and AT_2_ receptor immunoreactivity in the smooth muscle cell layer. **D**. Expression of AT_1_ and AT_2_ protein levels in human cerebral arteries incubated for 48 h with or without the MEK1/2 inhibitor U0126 (5 μM) and control human arteries. Data are expressed as percentage of fresh and given as mean ± s.e.m. *P<0.05, ** *P*<0.01.

In cultured arteries Ang II induced a concentration-dependent contraction with an E_max_ of 43 ± 15% and a pEC_50_ of 9.15 ± 1.65. These values were significantly higher than those observed in control arterial segments, in which an E_max_ of 12 ± 2% of was observed (Figure 4A, Table 1). The presence of U0126 during the organ culture produced a significantly attenuated Ang II induced response, compared to the cultured arteries. Interestingly there was no significant difference in the contractile response between control arteries and cultured arteries treated with U0126 (Figure 4A, Table 1). In the presence of the AT_2_ receptor antagonist PD12319 there was a diminished contraction after organ culture compared to control arteries, suggesting that the AT_2_ receptors are responsible for the upregulated responses induced by organ culture (data not shown).

#### Protein expression examined with immunohistochemistry

Immunohistochemistry showed a decrease in AT_1_ receptor protein in the smooth muscle cells after organ culture (62 ± 4%) as compared to control (100 ± 2%). Treatment with the MEK1/2 inhibitor U0126 prevented the down regulation of AT_1_ (82 ± 7%), receptor protein levels in the smooth muscle cell layer as compared to the organ culture (Figure 4B and 4D). The AT_2_ receptor protein was increased after organ culture (119 ± 2%) as compared to control (100 ± 2%). Incubation with U0126 prevented the increased expression of AT_2_ (111 ± 3%) receptor protein on the smooth muscle cells, however not significantly (Figure 4C-D, Table 2).

### 5-HT_1B_ receptor

#### Contractile response

**Figure 5:**
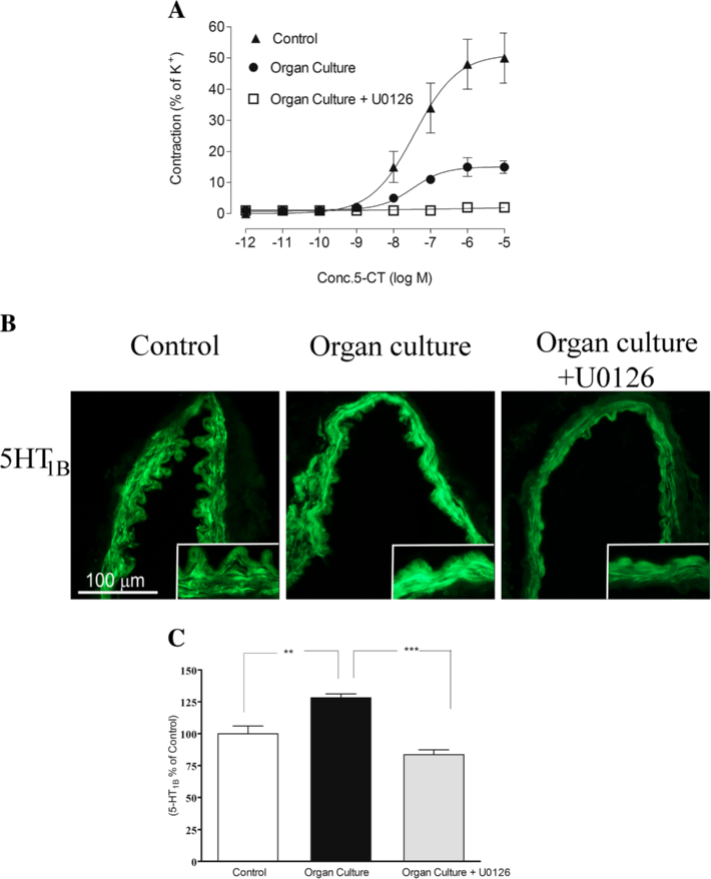
**A. Concentration response curves elicited by cumulative application of 5-CT in human cerebral arteries incubated for 48 h, human cerebral arteries incubated for 48 h with the MEK1/2 inhibitor U0126 (5 μM) and control human arteries.** Data are expressed as mean ± s.e.m. **B**. Sections from the human cerebral artery showing 5-HT_1B_ receptor immunoreactivity in the smooth muscle cell layer. **C**. Expression of 5-HT_1B_ protein levels in human cerebral arteries incubated for 48 h with or without the MEK1/2 inhibitor U0126 (5 μM) and control human arteries. Data are expressed as percentage of fresh and given as mean ± s.e.m. *P<0.05, ** *P*<0.01.

In cultured arteries 5-CT yielded significantly lower contractions that those observed in control arterial segments, presence of U0126 during the organ culture produced a significantly attenuated 5-CT induced response, compared to the cultured arteries (Figure 5A).

#### Protein expression examined by immunohistochemistry

The 5-HT_1B_ receptor protein was expressed in the smooth muscle cells and this signal was increased in organ culture (128 ± 3%) as compared to control (100 ± 6%). Treatment with the MEK1/2 inhibitor U0126 prevented the upregulation of 5-HT_1B_ (84 ± 4%), receptor protein levels in the smooth muscle cell layer as compared to the organ culture (Figure 5B-C, Table 2).

### pERK1/2

#### Protein expression examined by immunohistochemistry

**Figure 6:**
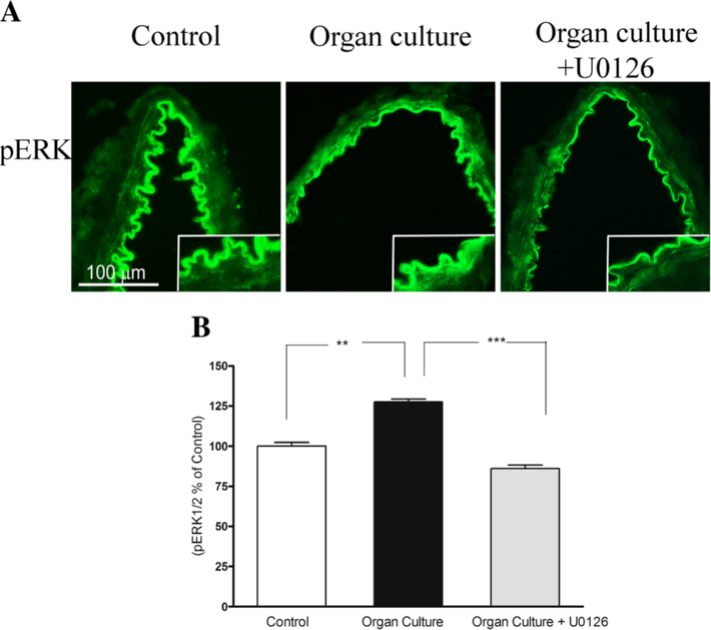
**A. Sections from the human cerebral artery showing pERK immunoreactivity in the smooth muscle cell layer.** There are increased expressions of the ERK1/2 protein levels in the cultured arteries compared to the control segments. Treatment with U0126 prevented the increased protein expression in the smooth muscle cells. **B**. Expression of pERK1/2 protein levels in human cerebral arteries incubated for 48 h with or without the MEK1/2 inhibitor U0126 (5 μM) and control human arteries. Data are expressed as percentage of fresh and given as mean ± s.e.m. *P<0.05, ** *P*<0.01.

The pERK1/2 protein was expressed in the smooth muscle cells and this signal was increased in organ culture (128 ± 2%) as compared to control (100 ± 2%). Treatment with the MEK1/2 inhibitor U0126 prevented the upregulation of pERK1/2 (86 ± 2%), protein levels in the smooth muscle cell layer as compared to the organ culture (Figure 6A-B).

## Discussion

This study demonstrates that there is a clear association between human cerebrovascular receptor upregulation via transcription involving activation of the MAPK pathway after organ culture. This was shown by the close association between pERK1/2 activation and the enhanced expression of the contractile receptors at both protein level and at a functional level.

The study design was based on previous work that has shown that organ culture induces upregulation of cerebrovascular receptors in a manner similar as that observed in experimental SAH and MCAO [36,37]. Organ culture is not a model for stroke, however, changes in vasoconstrictor responses after in vitro organ culture show a remarkable similarity to changes observed in animal models of ischemic and hemorrhagic stroke, which makes organ culture a useful in vitro method to study the pharmacological characteristics and underlying molecular and cellular mechanism of cerebrovascular receptor alterations.

In man cerebral vessels after a stroke contains elevated levels of several cerebrovascular receptor types ET_A_, ET_B_, AT_1,_ AT_2_ and 5-HT_1B_[38] and hence is in agreement with the experimental data obtained in animals. The present study revealed several novel and important findings: (i) While the contractile responses to ET-1 usually means activation of the ET_A_ receptors we found that there is an interaction between the ET_A_ and ET_B_ receptors in cerebral arteries following SAH both in experimental studies and in organ culture of human brain vessels [19,35]. The present immunohistochemistry provide experimental evidence that both ET_A_ and ET_B_ receptors are upregulated after organ culture. (ii) The angiotensin II responses were increased. Contrary to animal data the responses were unaltered by specific angiotensin AT_1_ blockers [22]. In animal models of stroke an upregulation of the contractile response to Ang II is observed and this contraction is mediated by the AT_1_ receptor [22,36]. In our study in the human arteries the results showed that in presence of the AT_2_ receptor antagonist PD12319 there was a diminished contraction after organ culture compared to control arteries, suggesting that the AT_2_ receptors are responsible for the upregulated responses induced by organ culture. Since the immunocytochemistry revealed that it was only the AT_2_ receptor protein that was elevated in the cerebral artery smooth muscle cells the evidence suggests that there is de novo upregulation of AT_2_ receptors producing contraction in the human brain arteries after organ culture. (iii) The 5-HT responses were reduced by organ culture, a finding in concert with what we have seen in tMCAO using two different models as well as after 24 h of organ culture.

Although the decrease in 5-HT_1B_-mediated contractile responses are less pronounced after organ culture than after experimental stroke [37]. In the subarachnoid hemorrhage model and global ischemia we have demonstrated an upregulation of the 5-HT_1B_ receptor [20,39]. Hence, whereas smooth muscle ET_B_ receptors are upregulated after all types of cerebral ischemia investigated so far (subarachnoid hemorrhage, global cerebral ischemia, and focal cerebral ischemia) as well as after organ culture, changes in the expression of the 5-HT_1B_ receptor appear to vary with the type of cerebral ischemia. The role of 5-HT and its receptor in ischemia are not clear; while some studies report a protective role for 5-HT receptor agonists, others show increased contractility and improvement with 5-HT receptor antagonist [40,41]. The results from the study of TP receptors revealed an increased response to the agonist but no significant upregulation of receptor protein. In experimental SAH a similar type of reaction appears [24]. The GPCR changes that we have observed in the human cerebral arteries after organ culture show a striking similarity to the changes observed in experimental cerebral ischemia and thus complements the picture of reactions.

Early during organ culture the raf-MEK-ERK pathway is activated and remains activated during the first two days of culture [32]. Other MAPK members such as p38 and JNK may also be activated but this seems to occur later during organ culture. Detailed study of major cerebral arteries and intracerebral microvessels were performed following experimental SAH [25]; the results clearly demonstrated that the MEK/ERK1/2 pathway was activated within minutes and remained activated until end of the 48 h period. On the other hand p38 and JNK reached significance only at 48 h. This is a model of SAH, however, a similar phenomenon was observed after MCA occlusion for 2 h and then reperfusion for 48 h both in large cerebral arteries and in microvessels within the brain tissue [42]. In the present study we verified that organ culture results in enhanced expression of pERK1/2 in the smooth muscle cells. Co-administration of the specific MEK1/2 inhibitor U0126 abolished this, confirming that the MEK/ERK pathway is important.

In cultured human arteries, the specific blockade of the MAPK MEK1/2 activity abolished the vascular smooth muscle cell receptor upregulation. A number of mechanisms and receptors have been proposed to account for the late cerebral ischemia [43] but no drug exist with good effect. Clazosentan, an endothelin receptor antagonist, was in a recent clinical study shown to result in reduction in vasospasm as seen angiographically but the outcome was not altered [44]. This was taken as evidence that we may consider also other events than just arterial narrowing; early brain injury and cortical spreading depression. We propose that cerebrovascular receptor upregulation may be such a mechanism that could be of importance, the current study reveals a mechanism present in man that can be modified with inhibition of raf-MEK-ERK signaling.

## Conclusion

In conclusion, we show that specific inhibition of the MAPK pathway using U0126 significantly attenuates the vasoconstriction mediated by ET, AT and TP receptors in human cerebral arteries and the enhanced expression of their receptors. The results indicate that MAPK inhibition might be a novel target for treatment of cerebrovascular disorders.

## Authors’ contributions

SA participated in the design of the study, guided the experimental procedures analyzed the data, and wrote the manuscript. SE performed the immunohistochemistry and prepared the images for it and reviewed the manuscript. RW performed the intensity measurements and reviewed the manuscript. EN carried out the organ culture and in vitro pharmacology myograph experiments. ON and HS performed the surgeries and reviewed the manuscript. LE conceived the study and participated in writing the manuscript. All authors read and approved the final manuscript.
